# Continuous versus intermittent nebulization of salbutamol in acute Severe asthma in children under 12 years of age

**DOI:** 10.12669/pjms.42.1.11935

**Published:** 2026-01

**Authors:** Arif Zulqarnain, Ayesha Fayyaz, Sulman Zafar, Beenish Fatima

**Affiliations:** 1 Arif Zulqarnain, FCPS. Department of Pediatric Medicine, Tertiary Care Hospital Nishtar 2, Multan, Pakistan; 2 Ayesha Fayyaz, FCPS. Department of Pediatric Medicine, The Children’s Hospital and The Institute of Child Health, Multan, Pakistan; 3 Sulman Zafar, FCPS. Department of Pediatric Medicine, Tertiary Care Hospital Nishtar 2, Multan, Pakistan; 4 Beenish Fatima, FCPS. Department of Pediatric Medicine, Tertiary Care Hospital Nishtar 2, Multan, Pakistan

**Keywords:** Asthma, children, Length of stay, Pakistan, Safety

## Abstract

**Objective:**

To compare the efficacy of continuous versus intermittent nebulization of salbutamol (SB) in the treatment of acute severe asthma (ASA) in children visiting the emergency department.

**Methodology:**

This randomized controlled trial was performed at the emergency department of pediatric medicine, The Children’s Hospital and The Institute of Child Health, Multan, Pakistan, during April 2024 to September 2024. Children aged 2-12 years, diagnosed with severe exacerbation of acute asthma, with clinical asthma score (CAS) > 8. Group-A was administered continuous SB nebulization at 0.3 mg/kg/hour for four hours, with 5-15 mg/hour. In Group-B, SB was administered intermittently at a dose of 0.15 mg/kg per dose. CAS, length of hospital stay, and treatment duration, were compared.

**Results:**

In a total of 120 children, 68 (56.7%) were male. The mean age was 8.06±1.6 years. Between Group-A (Continuous), and Group-B (Intermittent), there were no significant differences at 20 minutes (p=0.673), 40 minutes (p=0.419), one-hour (p=0.365), two-hours (p=0.536), and four-hours (p=0.536) in terms of CAS. The time to treatment initiation (p=0.837), time to treatment stop (p=0.77), duration of treatment (p=0.084), and hospital stay duration (p=0.959) were statistically similar in both treatment groups. Both groups reported relatively higher safety profile (95.0% [n=57] patients without any side effects in Group-A vs. 96.7% [n=58] in Group-B, p=0.648).

**Conclusion:**

Continuous, or intermittent nebulization of SB were both effective methods for treating severe exacerbation of acute asthma in children. CAS, treatment duration, or length of hospital stay between the two methods did not exhibit any significant differences.

***Trial Registration:*** NCT06754631 (ClinicalTrials.gov).

## INTRODUCTION

Asthma involves inflammation that affects the airways, and leads to bronchial hyperreactivity, narrowing of airways, structural changes in airway walls, and increased production of mucus.^1^ Asthma remains an important public health issue, significantly affecting the quality of life for many affected individuals. Asthma is a major global health issues, affecting around 300 million people, and causing about 1,000 deaths daily.^2^ Asthma affects around 260 million people globally, causing around 0.5 million deaths annually.^3^ The global mortality rate for pediatric asthma varies, ranging from 0.1-0.7 deaths/100,000.^4^

The most frequently observed symptoms among children experiencing asthma include flare-ups, chest tightness, wheezing, and shortness of breath.^5^,^6^ Salbutamol (SB) is commonly the 1^st^ treatment given in acute severe asthma (ASA), and researchers have shown it to be safer and effective approach for alleviating symptoms.^7^,^8^ While it was once reserved for continuous nebulization only in life-threatening situations, it is now also routinely used in pediatric intensive care units (PICUs).^9^

Continuous use of SB inhalation has been shown to be more effective than sporadic administration.^7^ Nebulized SB, in particular, is recognized as a safer and more effective method for alleviating symptoms of severe asthma.^10^ During ASA attack, healthcare providers typically administer nebulized SB intermittently, ranging from 1-4 hours.^11^ The ongoing debate regarding the comparative effectiveness and safety of continuous versus intermittent nebulization serves as the foundation for the current study. There is lack of local pediatric emergency department data from South Punjab and uncertainty about safety if continuous versus intermittent nubulization of SB, especially in resource-limited settings. It was hypothesized that there is a significant difference in terms of CAS following continuous versus intermittent nebulization of Salbutamol in acute severe asthma in children. This study aimed to compare the effects of continuous versus intermittent nebulization of SB in the treating severe exacerbation of ASA in children visiting the emergency department (ED) of a tertiary childcare hospital of South Punjab, Pakistan. By exploring this comparison, this study seeks to improve the management of ASA attacks in the emergency room and may contribute to reducing the incidence and severity of such episodes in pediatric patients.

## METHODOLOGY

This open labeled, randomized controlled trial was conducted at the ED of pediatric medicine, The Children’s Hospital and The Institute of Child Health, Multan, Pakistan, during April 2024 to September 2024.

### Ethical Approval.

It was obtained from “Institutional Ethical Committee” was obtained prior to the commencement of this study (letter number: 2425, dated: February 2, 2024). Informed and written consents were acquired from the parents/guardians of children participating in this study. Trial registration was done as NCT06754631 (ClinicalTrials.gov). A sample size of 120 (60 in each group) was calculated taking the anticipated proportion of success in continuous, and intermittent nebulization as 87.5%, and 57.1%,^12^ with 95% confidence interval and 95% power of the study.

### Inclusion & Exclusion Criteria:

The inclusion criteria comprised of children aged 2-12 years, with severe exacerbation of acute asthma based on the clinical asthma score (CAS) > 8. Exclusion criteria included children who were treated with other treatment options. Children at imminent risk of respiratory arrest, or those with congenital heart disease, chronic respiratory conditions, or neurological disorders (as indicated by medical history and records), were excluded. Children who required mechanical ventilation prior to enrollment were also excluded. Children referred from other hospitals with no available data on emergency treatments received were also not included. Simple random sampling technique was adopted.

Children were randomly allocated to either Group-A (continuous) or Group-B (intermittent). Children in both study groups were given same total amount of albuterol via nebulization over four hours. Group-A was administered continuous SB nebulization at 0.3 mg/kg/hour for four hours, with 5-15 mg/hour, administered via “High Output Extended Aerosol Respiratory Therapy (HEART)” through a face mask at an oxygen flow rate of 10 L/min. In Group-B, SB was administered intermittently at a dose of 0.15 mg/kg per dose via a face mask with an oxygen flow rate of 6-8 L/min. Group-B received 2-3 nebulizations, with no more than 7.5 mg per dose, every 30 minutes for four hours. The study utilized a jet nebulizer system (Model: OMRON NE-C28P, Omron Healthcare Co. Ltd., Japan), which produces particles with a mass median aerodynamic diameter of approximately 3 µm, suitable for lower airway deposition in pediatric asthma management. A well-fitted pediatric face mask was used to minimize aerosol loss and ensure effective delivery. All nebulizations were administered using a continuous oxygen flow rate of 10 L/min, as per institutional emergency protocol for severe asthma exacerbations. This flow rate was selected to ensure optimal aerosol generation and consistent medication delivery, especially in patients with increased respiratory effort. Lower or intermittent flow rates (e.g., 6–8 L/min) may reduce aerosol output and deposition efficiency, therefore, maintaining a continuous 10 L/min flow was aimed to achieve adequate bronchodilator delivery and faster clinical response in this critically ill population. The study was designed to aim that both groups received an equivalent total dose of salbutamol over the 4-hour treatment period. Continuous group received continuous nebulization at 0.3 mg/kg/hour × 4 hours = 1.2 mg/kg total dose, delivered with an oxygen flow rate of 10 L/min. Intermittent group received intermittent nebulization at 0.15 mg/kg per dose every 30 minutes for 8 doses = 1.2 mg/kg total dose, delivered with an oxygen flow rate of 6–8 L/minutes.

To minimize allocation bias, the random sequence was generated using a computer-based random number generator by an independent statistician who had no role in patient recruitment or assessment. Allocation concealment was ensured through sequentially numbered, opaque, sealed envelopes, which were opened only after participant enrollment by a study coordinator not involved in outcome evaluation. All children received standard supportive treatment, which included supplemental oxygen therapy to maintain SpO_2_≥95%, adequate intravenous hydration, and appropriate intravenous medications such as systemic corticosteroids and bronchodilators, administered according to institutional emergency protocols.

**Fig.1:**
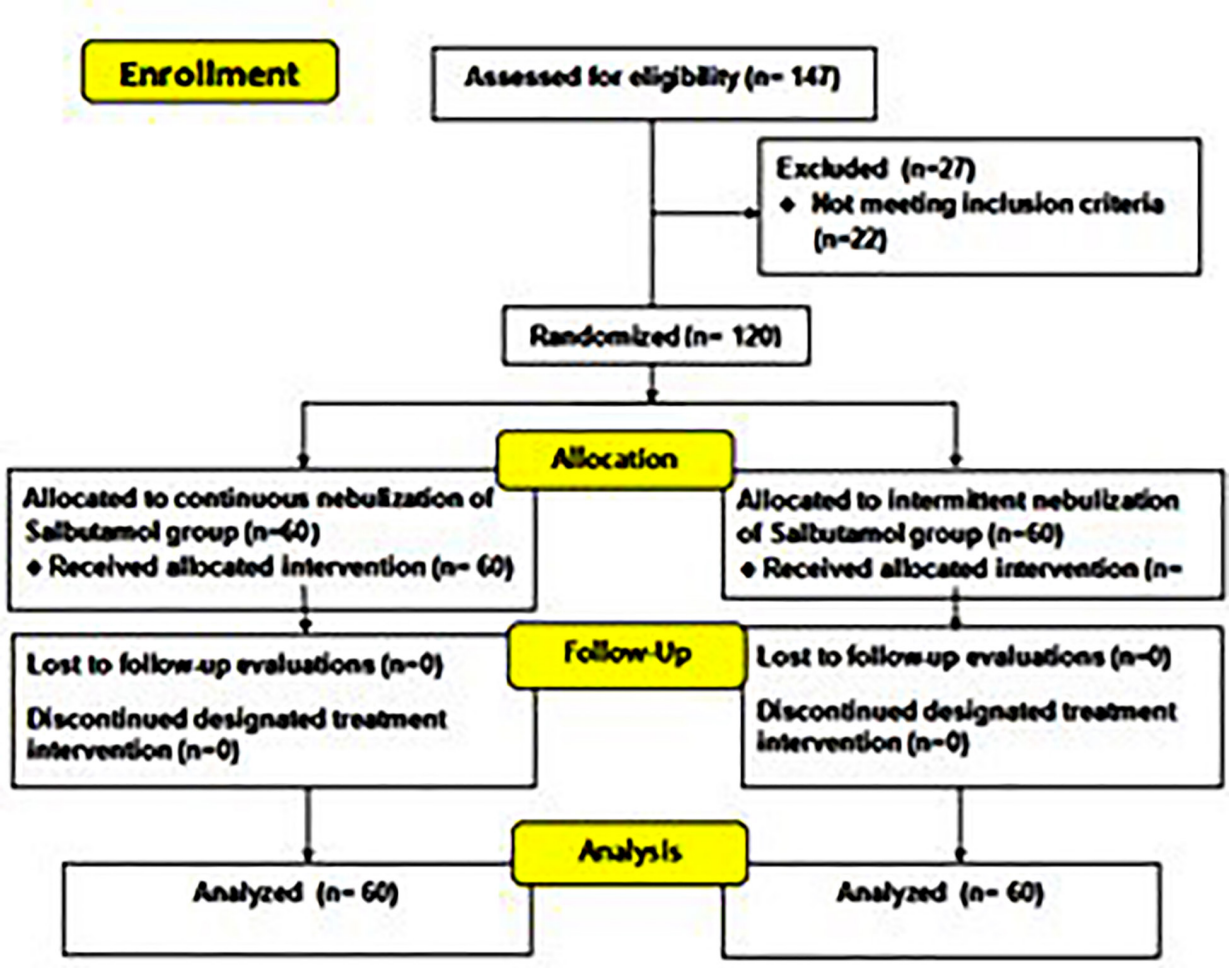
CONSORT flow diagram.

The children were treated in the emergency room for up to four hours and monitored for an additional two hours. Re-evaluations occurred at 20-minutes, 40-minutes, one-hour, two-hours, and four-hours after treatment initiation (Fig.1). The CAS was evaluated at baseline, and at each re-evaluation point up to four hours. The CAS includes evaluation of respiratory rate, wheezing, indrawing, dyspnea, and inspiratory to expiratory (I:E) ratio (each scored from 0 to 3).^13^ A score of 0-4 is considered mild, 5-8 as moderate, and > 8 as severe.^14^ Although, CAS has not been formally validated in Pakistani children, it has demonstrated strong inter-rater reliability of 0.82 (weighted kappa), and discriminatory reliability of 0.92 (Ferfuson’s delta).^15^ The CAS for each patient was assessed by a trained pediatric resident who was part of the emergency department team. To minimize observer bias, the same resident performed all evaluations for a given participant. All residents were oriented and standardized in the use of the CAS before study initiation through a structured training session conducted by the supervising consultant. Assessments were based strictly on predefined criteria for each score component of CAS. Admission, treatment duration, and discharge times were also tracked. Safety was monitored by ensuring that no unfavorable effects, such as nausea, vomiting, tremors, flushing, dizziness, or tachycardia, occurred in the enrolled patients. Safety monitoring was performed throughout the 4-hour nebulization period. Heart rate, respiratory rate, and SpO_2_ were recorded at baseline and at 20, 40, 60, 120, and 240 minutes, with continuous pulse oximetry. Tachycardia was defined as HR >160 bpm (2–5 years), >140 bpm (6–10 years), or >120 bpm (>10 years). To minimize bias, all outcome assessments were based on objective, predefined clinical criteria and standardized scoring systems rather than subjective judgment. Data collectors were trained uniformly, and outcome variables were verified through chart review using standardized case report forms.

### Statistical analysis:

For data analysis, “IBM-SPSS Statistics, version 26.0” was used. Qualitative data were expressed as frequency and percentages. Quantitative data were shown as mean and standard deviation (SD), or median and interquartlie range (IQR), depending upon the normality distribution of the data assessed by Shapiro-Wilk test. Between groups, the independent t-test was used to compare continuous data, while chi-square or fisher’s exact test was applied for categorical data. Repeated measures analysis of variance was applied to assess changes in CAS scores over time within groups. P<0.05 was taken as statistically significant.

## RESULTS

In a total of 120 children, 68 (56.7%) were male, and 52 (43.3%) female. The mean age was 8.06±1.6 years, while 89 (74.2%) children were aged below 10 years. There were 76 (63.3%) children who belonged to rural areas of residence. Demographic characteristics were comparable between the study groups (Table-I).

**Table-I:** Characteristics of children in both study groups.

Demographics		Total	Group-A	Group-B	P-value
Gender	Male	68 (56.7%)	33 (48.5%)	35 (51.5%)	0.727
	Female	52 (43.3%)	27 (51.9%)	25 (48.1%)
Age (years), Mean±SD		8.06±1.6	8.11±1.8	7.98±1.5	0.668
Age groups (years)	<10	89 (74.2%)	42 (47.2%)	47 (52.8%)	0.297
	≥10	31 (25.8%)	18 (58.1%)	13 (41.9%)
Weight (kg), Mean±SD		25.09±4.2	25.15±4.5	25.06±3.9	0.907
Weight (kg)	<30	91 (75.8%)	44 (48.4%)	47 (51.6%)	0.522
	≥30	29 (24.2%)	16 (55.2%)	13 (46.8%)	
Residence	Urban	44 (36.7%)	24 (54.5%)	20 (45.5%)	0.449
	Rural	76 (63.3%)	36 (47.4%)	40 (52.6%)	

**Fig.2:**
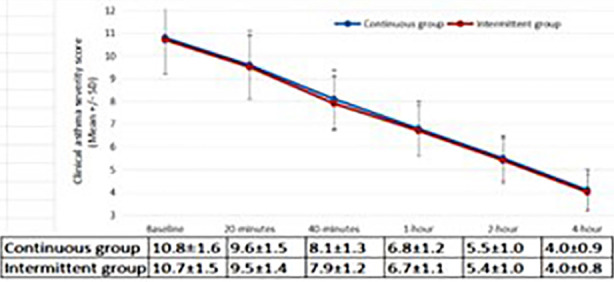
Comparison of clinical asthma score in both treatment groups during study intervals.

**Fig.3:**
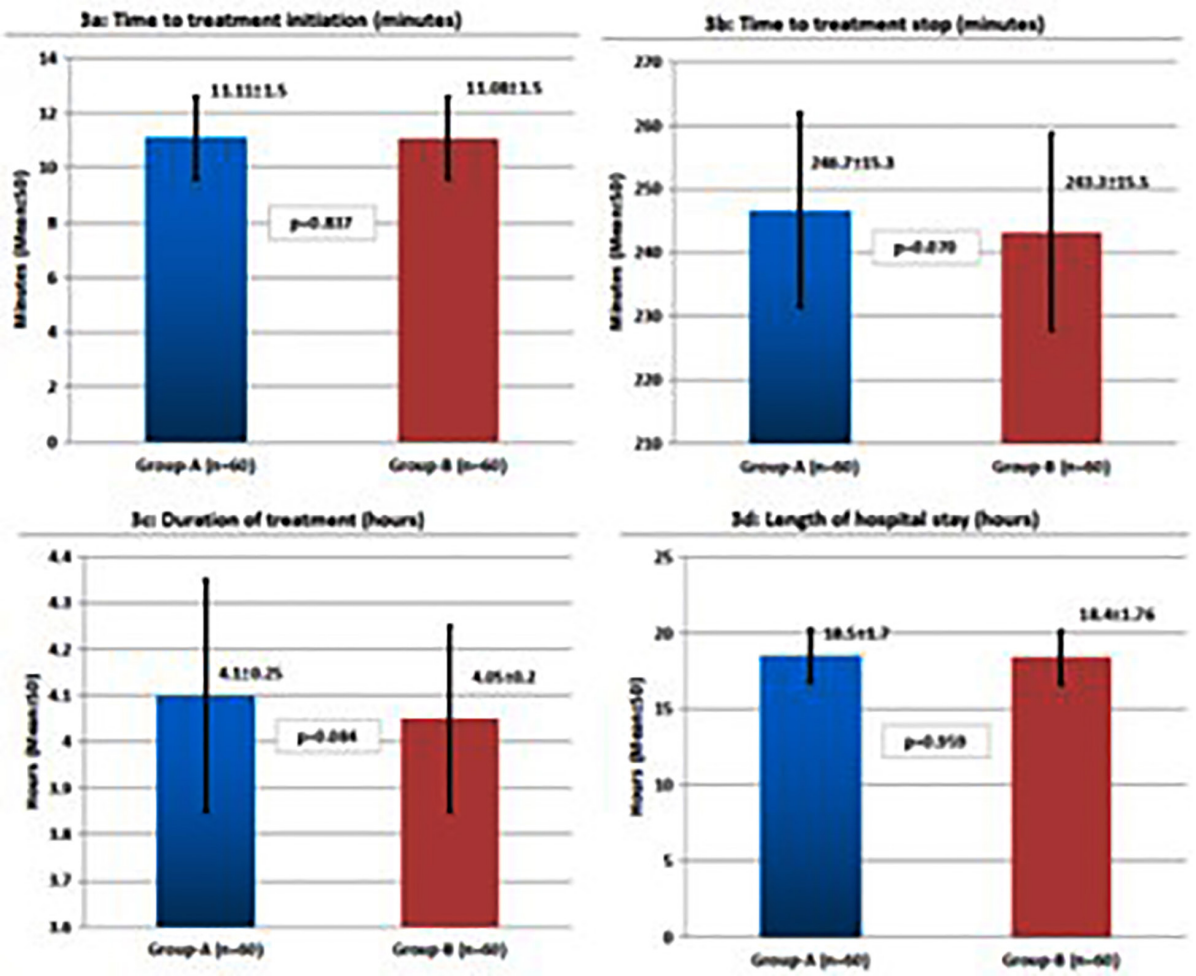
Comparison of time to treatment initiation (3a), treatment stop (3b), duration of treatment (3c), and length of hospitalization (3d) in both treatment groups.

At baseline, CAS was 10.8±1.6 in the continuous nebulization group, and 10.7±1.5 in the intermittent group (p=0.749). At 20 minutes, the mean CAS was 9.6±1.5 vs. 9.5±1.4 (p = 0.673), 8.1±1.3 vs. 7.9±1.2 (p = 0.419) at 40 minutes, 6.8±1.2 vs. 6.7±1.1 (p = 0.536) at one hour, 5.5±1.0 vs. 5.4±1.0 (p = 0.536) at two hours, and 4.1±0.9 vs. 4.0±0.8 (p = 0.536) at four hours, for the continuous and intermittent groups, respectively (Fig.2). On within-group analysis, there was statistically significant reduction in CAS over time in both groups (p<0.001). Both the groups were comparable (p>0.05) on time to treatment initiation (Fig.3a), time to treatment stop (Fig.3b), duration of treatment (Fig.3c), and hospital stay (figure-3d). Both groups reported relatively higher safety profile as no side effects were reported in 57 (95.0%) patients in Group-A vs. 58 (96.7%) in Group-B, p=0.648).

## DISCUSSION

This study demonstrated that CAS at multiple time points between the two SB nebulization methods was statistically similar. Both approaches showed comparable improvement in CAS over time, with no significant difference in the duration of treatment, time to initiation, or length of hospital stay. Besbes-Ouanes et al.,^16^ compared the clinical effects of continuous versus intermittent nebulization in ASA. Their findings were consistent with ours, showing no statistical differences in clinical outcomes. This similarity underscores the idea that while continuous nebulization may be intuitively more effective due to its constant administration, intermittent nebulization with more frequent doses could achieve similar clinical results.^17^ Kulalert et al.,^12^ compared the two treatment modalities using a retrospective cohort study and found that continuous nebulization offered a higher proportion of success.

The findings of the present study showed that both methods led to comparable improvements in CAS is in agreement with a local study reinforcing the conclusion that either nebulization strategy could be effective in treating acute severe asthma exacerbations in children.^16^ Khine et al., further supports our findings, where no significant differences were found between the continuous and intermittent nebulization methods in terms of treatment success or hospitalization rates.^18^ Khine et al,^18^ like this study showed that the time spent on nebulization therapy and the clinical outcomes, such as the CAS and the need for hospitalization, were statistically similar in between both treatment approaches.^19^,^20^

The findings of our study are important because these suggest that intermittent nebulization may be a viable alternative to continuous nebulization in managing severe asthma exacerbations. The similar clinical outcomes in both groups, as shown by the comparable CAS at multiple time points, indicate that either treatment strategy can be used without compromising the quality of care. The shorter time commitment for healthcare providers associated with intermittent nebulization may lead to more efficient use of resources in the busy pediatric emergency department setting.^21^

The intermittent nebulization might be more feasible and cost-effective in settings with limited resources, whereas continuous nebulization, while effective, requires dedicated equipment such as high-output nebulizers, and it may demand more time and attention from healthcare providers. Intermittent nebulization, on the other hand, can be delivered using standard nebulizer equipment and may allow for quicker turnover in a clinical setting.^18^ This efficiency could improve patient flow in emergency departments, particularly in high-volume settings where rapid management of asthma exacerbations is essential.

Both groups exhibited high safety profiles, with no major adverse effects observed, and only a minimal difference in safety outcomes (95.0% vs. 96.7%). The absence of any major adverse effects of SB reinforces the safety of both treatment approaches.^22.23^ Besbes-Ouanes et al., reported low rates of treatment failure and adverse effects, further supporting the notion that both continuous and intermittent nebulization are safe treatment options for children with acute severe asthma.^16^

### Limitations

Despite the promising findings, our study has several limitations. One limitation is the relatively short duration of the monitoring period. Although we followed patients for six hours after the initiation of treatment, longer-term outcomes, such as asthma control in the weeks following the exacerbation, were not assessed. It is possible that the efficacy of nebulization methods may differ in the long term, particularly in terms of preventing future exacerbation. Being a single center study, these findings cannot be generalized. The pathophysiology and clinical presentation of asthma may differ in older populations, and therefore, the efficacy and safety of nebulization techniques could vary. Device differences and delivery efficiency may have confounded results.

## CONCLUSION

This study provides evidence that continuous and intermittent nebulization of SB are both effective and safe methods for treating ASA exacerbations in children. CAS, treatment duration, or length of hospital stay between the two methods were relatively similar. This could be particularly beneficial in settings with limited resources or high patient volumes.

### Authors contribution:

**AZ:** Conception and design, drafting, proof reading, critical revie.,

**AF:** Data collection, responsible for data’s integrity, proof reading, critical review,

**SZ & BF:** Data synthesis and analysis, drafting, proof reading, critical review

All author shave read and approved the final version and are accountable for the integrity of the study.
